# A simple technique can reduce cardiopulmonary bypass use during lung transplantation

**DOI:** 10.6061/clinics/2016(04)10

**Published:** 2016-04

**Authors:** Marcos N Samano, Leandro R Iuamoto, Hugo V S Fonseca, Lucas M Fernandes, Luis G Abdalla, Fabio B Jatene, Paulo M Pêgo-Fernandes

**Affiliations:** Instituto do Coração (InCor) do Hospital das Clínicas da Faculdade de Medicina da Universidade de São Paulo, Divisão de Cirurgia Torácica, São Paulo/SP, Brazil

**Keywords:** Lung Transplantation, Cardiopulmonary Bypass, Thoracic Surgery, Thoracic Surgical Procedures, Surgical Procedures, Operative

## Abstract

Cardiopulmonary bypass causes an inflammatory response and consumption of coagulation factors, increasing the risk of bleeding and neurological and renal complications. Its use during lung transplantation may be due to pulmonary hypertension or associated cardiac defects or just for better exposure of the pulmonary hilum. We describe a simple technique, or *open pericardium retraction*, to improve hilar exposure by lifting the heart by upward retraction of the pericardial sac. This technique permits lung transplantation without cardiopulmonary bypass when bypass use is recommended only for better exposure.

## INTRODUCTION

Since the development of bilateral sequential lung transplantation (LTx), cardiopulmonary bypass (CPB) has become unnecessary in most cases. However, in cases of severe pulmonary hypertension, associated cardiac defects and ventilatory and hemodynamic decompensation, its use is still obligatory. Difficulty in exposing the left pulmonary hilum is another situation in which CPB is recommended. Although CPB use may have a protective effect by reducing blood flow to the newly implanted lung, its use is associated with an inflammatory reaction, coagulation disturbances, acute alveolar injury and renal and neurological complications. There are many reports regarding this topic, but there is no consensus on the benefit of CPB during LTx, and its use depends on the transplantation team's familiarity with CPB. We describe a simple technique, or *open pericardium retraction*, to aid exposure of the pulmonary hilum; this method could be used to avoid CPB in most cases.

## MATERIALS AND METHODS

Our standard approach to bilateral LTx is bilateral anterolateral thoracotomies with a sternum “V” shape division (“clamshell” incision). For patients with large pleural cavities, as is the case in chronic obstructive pulmonary disorder (COPD) and cystic fibrosis, the sternum division may not be necessary; however, there is no reason to spare the sternum in patients with pulmonary fibrosis and small pleural cavities or an asymmetric thorax. Once the chest is opened, the first lung to be removed is the one with the lowest perfusion. We first try to release all pulmonary adhesions and dissect the hilum bilaterally, after which we start the pneumonectomy. When the chest cavity is too tight due to an elevated diaphragm, one or two stitches applied to the apex of the diaphragmatic dome and pulled through the chest tube incision should be sufficient to increase the space. The side with the lowest perfusion is usually on the left, but the heart's position makes the left hilum harder to expose. However, this issue depends on the underlying disease because in certain cases, there is severe retraction of the lungs to the right, so the right hilum becomes the hardest to expose.

**Figure 1:**
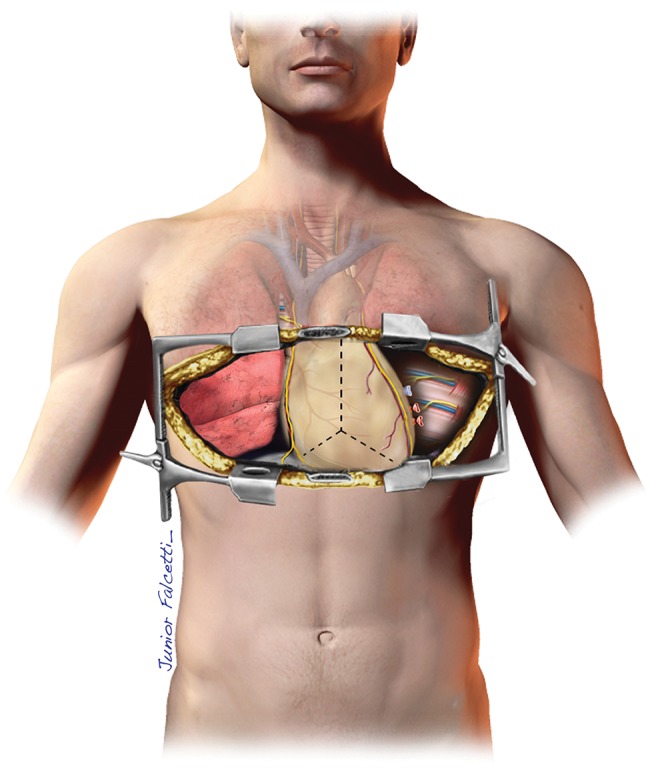
Left view of the chest cavity from the “clamshell” incision, without retraction of the pericardium. It is not possible to visualize the left atrium or the pulmonary veins.

**Figure 2:**
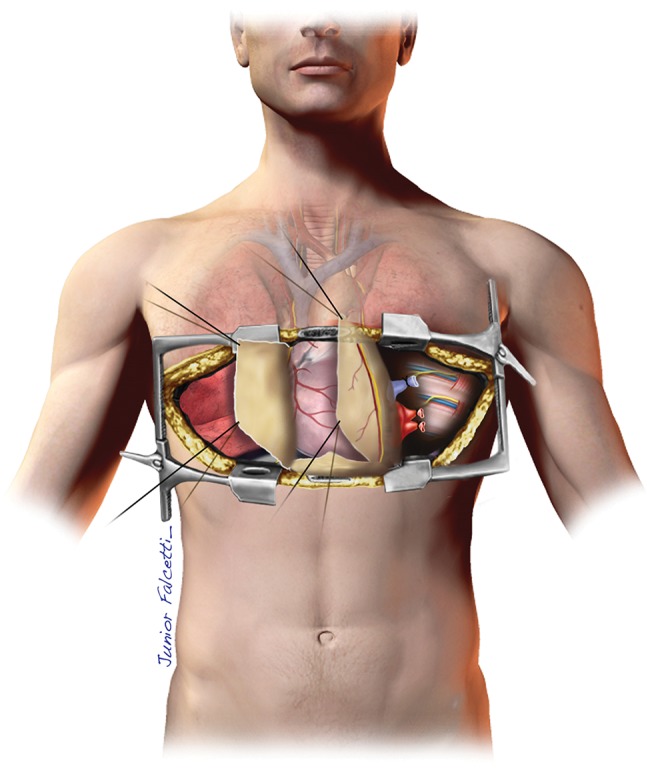
The same view with retraction of the pericardium. The upper and lower pulmonary veins are now visible.

The pericardial sac is opened with a vertical midline incision and is laterally extended downward in a “Y” shape. Sometimes, it is possible to observe tension relief of the heart with the opening of the pericardium. Three or four stitches are applied to each side of the pericardium, and the second assistant applies upward retraction of the ipsilateral stitches. This *open pericardium retraction* allows greater exposure of the inferior pulmonary ligament and inferior vein (Figures 1 and 2). Hemodynamic parameters should be continuously monitored because the lifting of the heart may cause a drop in blood pressure (BP). The pulmonary vessels are isolated, and the artery is cross-clamped to measure pulmonary pressure. If no significant changes in BP or arrhythmia occur, the artery is ligated and transected. However, in cases of mild pulmonary hypertension, clamping the artery may cause instability, and CPB may be necessary for hemodynamic reasons.

### The sequence of the anastomosis technique is the same for transplants with and without CPB: bronchus, artery and atrium.

The lung is inflated and the reperfusion is initiated by slowly declamping the artery. At this time, the left atrial anastomosis is intentionally kept open without tightening the suture, promoting removal of clots and air. The suture is completed after controlled bleeding, and the artery clamp removed after 10 minutes of reperfusion. The heart is then returned to its normal position, the pericardium is kept open, and two chest tubes are used in each side.

## COMMENT

The first successful single lung transplant was performed by Joel Cooper in Toronto in 1983, without CPB assistance 1. The en bloc bilateral technique described in 1988 was performed with mandatory CPB assistance and tracheal, pulmonary trunk and left atrial anastomoses 2. In contrast, the sequential bilateral technique described by the same group in 1990, involving the performance of two single lung transplants, showed that CPB was not always mandatory for bilateral procedures. However, thirty years after the first successful LTx, there is no consensus about CPB use in LTx.

Elective CPB makes LTx technically easier; promotes hemodynamic stability; and has a protective effect on the first implanted lung because during the sequential procedure, this lung receives all volemia, and a higher hydrostatic pressure can produce alveolar and severe interstitial edema. Moreover, oxygenation by CPB is nearly always stable and is not dependent on correct positioning of the double-lumen tube, which is a very difficult ventilation strategy in young adults and children 3. However, CPB also promotes an inflammatory response that is indistinguishable from ischemic-reperfusion injury in many cases. Bleeding and coagulopathy (consumptive and heparin induced), lung alveolar injury, renal dysfunction and neurological disorders are also related to CPB 4.

Studies concerning the use of CPB in LTx are lacking, and those available in the literature have a low level of evidence because they are retrospective observational studies. Additionally, there is no expectation of major studies on this topic in the future because the issue depends on several clinical factors and surgical team experience 5. There are two established strategies for CPB during LTx: programmed and nonprogrammed. Certain conditions relating to programmed use are well known, including pulmonary hypertension and associated congenital cardiac defects. Other conditions, such as ventilation disturbance, pulmonary hypertension decompensation, right ventricular dysfunction and hemodynamic instability during mediastinal retraction, are causes for non-programmed CPB.

Hemodynamic instability caused by mediastinal manipulation during transplantation is more common on the left side due to the heart's position. The left pulmonary veins are posterior, and liberation of the inferior pulmonary ligament and exposure of the left atrium could be extremely hazardous. For this reason, CPB is often used in this condition. Lau et al. described the use of a suction device, usually used for myocardial revascularization without CPB, for heart repositioning, with the intention of improving exposure and minimizing the instability caused by heart retraction 6. At that time, the authors successfully applied this technique in three cases, avoiding the use of CPB.

During certain cases of bilateral procedures, we have observed stretching of the anterior pericardial layer after thoracic retraction over a “clamshell” incision. The pericardium became tight, causing a drop in BP. In several situations, when the observed hypotension suggested use of CPB, the simple aperture of the pericardial cavity induced marked relaxation, a decrease in myocardial restriction and immediate improvement of BP. Based on these observational parameters, we applied 3 or 4 stitches over the free borders of the pericardium, and with a gentle *open pericardium retraction*, we were able to pull the heart out of its cavity without a reduction in BP. The posterior structures, such as those in the inferior ligament and inferior pulmonary vein, were then easily exposed and dissected. A comfortable view was acquired, allowing safe preparation of the left atrium and easy application of a vascular clamp for atrial anastomosis. Since 2010, we have successfully applied this technique, without the use of CPB, in situations in which exposure of the left hilum was difficult, as well as on the right side in cases of asymmetric thorax.

As conclusion, this maneuver can be useful in situations in which the pulmonary trunk is posteriorly hidden or in cases in which the thoracotomy induces pericardial restriction. Regardless, in *open pericardium retraction* in the case of hemodynamic instability, extracorporeal circulation is implanted in the conventional way, via central cannulation (right atrium and aorta).

## AUTHOR CONTRIBUTIONS

Samano MN was responsible for the study design, manuscript writing, analysis of the technique, manuscript revision and figure elaboration. Iuamoto LR, Fonseca HV and Fernandes LM were responsible for the manuscript writing, analysis of the technique, paper revision and figure elaboration. Abdalla LG was responsible for the manuscript revision and figure elaboration. Jatene FB was responsible for the supervision of the study, teaching the technique and manuscript revision. Pêgo-Fernandes PM was responsible for the supervision of the study, teaching the technique and manuscript revision.
